# Supplementary material for: Rapid turnover of life-cycle-related genes in the brown algae
Source: Genome Biol. 2019 Feb 14;20:35. doi: 10.1186/s13059-019-1630-6 (PMC6374913; doi:10.1186/s13059-019-1630-6)
Supplement: Supplementary file 3 — Protein alignments, corresponding Gblocks html file and CDS sequences for the lineage conserved gametophyte-biased genes. (ZIP 2109 kb) [file 13059_2019_1630_MOESM3_ESM.zip › Con_GA_alignments/Ec-21_005630.aa_ali.fasta-gb.htm]

Ec-21\_005630.aa.fasta.trans.aa\_ali.fasta


## Gblocks 0.91b Results

Processed file: **Ec-21\_005630.aa.fasta.trans.aa\_ali.fasta**  
Number of sequences: **2**  
Alignment assumed to be: **Protein**  
New number of positions: **285** (selected positions are underlined in blue)

```
                         10        20        30        40        50        60
                 =========+=========+=========+=========+=========+=========+
Ec-21_005630     MYDADNRDKDKEDRKKSDRPPTIRQDELQDTCDTTTSGAAHVLASDVSRLASRQSEVASE
MSTRG.41586.1    ------------------------------------------------------------
                                                                             


                         70        80        90       100       110       120
                 =========+=========+=========+=========+=========+=========+
Ec-21_005630     VLQEPSSEDARGTLPADPRTFHRKFPKVLEALAVLGPSVGLQAKAVGLMEEVVERTDHSR
MSTRG.41586.1    ------------------------------------------------------------
                                                                             


                        130       140       150       160       170       180
                 =========+=========+=========+=========+=========+=========+
Ec-21_005630     IDARIEARAARKGRIGRLAESLAEDAKLVAARHQHTAARMEVAPDPATARFVADDIRRLT
MSTRG.41586.1    ------------------------------------------------------------
                                                                             


                        190       200       210       220       230       240
                 =========+=========+=========+=========+=========+=========+
Ec-21_005630     LVQEKLAYRVQQFILTASGAVFNSHAGEDNPHSINNVQNATIDNGSKTAVSGSAATVSNA
MSTRG.41586.1    ------------------------------------------------------------
                                                                             


                        250       260       270       280       290       300
                 =========+=========+=========+=========+=========+=========+
Ec-21_005630     AAETNMSEASMKETTTTETAQGRLGRLAAAVVKELRSGVDIGSVAAARAQAIRRGSQAGR
MSTRG.41586.1    ------------------------------------------------------------
                                                                             


                        310       320       330       340       350       360
                 =========+=========+=========+=========+=========+=========+
Ec-21_005630     LADELRLIASGQAAASLALGDAGAQLIADAEAEKPIPREYALSRSSSTHDHNPLGRTHCG
MSTRG.41586.1    ------------------------------------------------------------
                                                                             


                        370       380       390       400       410       420
                 =========+=========+=========+=========+=========+=========+
Ec-21_005630     KLEEINCISEIDRGDEGDSRGKGPANAAAVVVGHNDNEHDVVVIAPETASIVPNGLAAGT
MSTRG.41586.1    ------------------------------------------------------------
                                                                             


                        430       440       450       460       470       480
                 =========+=========+=========+=========+=========+=========+
Ec-21_005630     GDEFGPRGPSLPPAAVSVAGSSMAIEMAAQEEEIAAASRIQSFLRRRRRQGRLGIVPGGL
MSTRG.41586.1    ------------------------------------------------------------
                                                                             


                        490       500       510       520       530       540
                 =========+=========+=========+=========+=========+=========+
Ec-21_005630     STERATVVPVLPSAANSSGELCNAGSSRSRATADWFVVALRSLRREVDNFLLGGTGVDVV
MSTRG.41586.1    ------------------------------------------------------------
                                                                             


                        550       560       570       580       590       600
                 =========+=========+=========+=========+=========+=========+
Ec-21_005630     RGGSSDDGSAAPSVSDLGENAGDSGSDEDIDKADGVDGDSDNDGDDTEPESSEDCPDYDW
MSTRG.41586.1    ---------------------------------------LHRV-----------------
                                                                             


                        610       620       630       640       650       660
                 =========+=========+=========+=========+=========+=========+
Ec-21_005630     PTFPPVPLRARAPSLYTLPPPSSGSLRRKPRDSSERAPGVPASKGPFTKLDPEVAARLNS
MSTRG.41586.1    -----------------------GDDLEEEAEANSQSPRTDARRGIFTKLDPEVAARLSA
                                                      #######################


                        670       680       690       700       710       720
                 =========+=========+=========+=========+=========+=========+
Ec-21_005630     ASSVFCLRASDVLTAFSPPPPPPSSSRLIASTATSSDSLPRSRPSAIPGTSAGREGTGGF
MSTRG.41586.1    ATSIFSLRAADVLAAFIPPTVTSSSCPVEPPAGLRNEKLSGTRPSTVPRAAGGTR--RGS
                 #########################             ###############     ##


                        730       740       750       760       770       780
                 =========+=========+=========+=========+=========+=========+
Ec-21_005630     RRLSSPEALRQRLRVEAQDASLAGLGRPWLLSSRAVSLQTRHKQEQQ-RSAFLRKKGNSG
MSTRG.41586.1    ANLSSPEALRRRLRTEAEDASQAGLGRPWLLCSRKTRPPTRHRLEQQQRSAFVRMEKSAR
                 ###############################################             


                        790       800       810       820       830       840
                 =========+=========+=========+=========+=========+=========+
Ec-21_005630     KPIGMWGGCGGIDQVRGKRRRTDAFGPLTPLGQRLARRYNAGVAARRRRIIGETDAFRAV
MSTRG.41586.1    AAHGHVRSL-GFKQARECRRRADFFGPWTPLGRRLAKRFNAGRAARRRCIRDETDAFRVR
                           ##################################################


                        850       860       870       880       890       900
                 =========+=========+=========+=========+=========+=========+
Ec-21_005630     HVGRQLRIGASLNALERVERALDARLSCAREATVSSAHIAELLDVVGRFVFSDSQLCGQR
MSTRG.41586.1    QVGRQLRSGAALNALEQVERALDVRLSLAREERVPPAHVSELLDFVGRFVFSDSQLREEP
                 ############################################################


                        910       920       930       940       950       960
                 =========+=========+=========+=========+=========+=========+
Ec-21_005630     TEAASTPSGGEAVEGIAPQRSRCRICQELLEDLRQKFPVVREQSSWGNETNPFCCDRRWQ
MSTRG.41586.1    AETRVESSGGAAQNDSDPERNRCLICQDLLEDLRRKFPVVGDQSSWMDDRDPFCCDRCWQ
                 ############################################################


                        970       980       990      1000      1010      1020
                 =========+=========+=========+=========+=========+=========+
Ec-21_005630     GSPRIGSPEWENNTITPTSTDDPNQDNANDGRGNDAYNMSLERVASTEGAEEAQSPPSAV
MSTRG.41586.1    NXPA--------------------------------------------------------
                 ###                                                         


                       1030      1040      1050      1060      1070      1080
                 =========+=========+=========+=========+=========+=========+
Ec-21_005630     VGTTGLRVRNGDDAGASYGGAAMSWPSPSENAGGKNCPRPGLLPPVSTPGSVEEDTPATG
MSTRG.41586.1    ------------------------------------------------------------
                                                                             


                       1090      1100      1110      1120      1130      1140
                 =========+=========+=========+=========+=========+=========+
Ec-21_005630     SVRCRRGAAGLMQSGAVVPGLERRGSKVGAKPPPKPDAVAAAFSAHLHGVDKYPLELARL
MSTRG.41586.1    ------------------------------------------------------------
                                                                             


                       1150      1160      1170      1180      1190      1200
                 =========+=========+=========+=========+=========+=========+
Ec-21_005630     CRFAQFLTENELLREAYEVILHILSSFTTSERDGISGKDNRGNYGNSNRGNEGKSRPATP
MSTRG.41586.1    ------------------------------------------------------------
                                                                             


                       1210      1220      1230      1240      1250      1260
                 =========+=========+=========+=========+=========+=========+
Ec-21_005630     PSSWSFSPLPALPTGLRGQLLVVAGRLAVATGRYPQAVRHVKEAVRTVTDNSTKAGRDTS
MSTRG.41586.1    ------------------------------------------------------------
                                                                             


                       1270      1280      1290      1300      1310      1320
                 =========+=========+=========+=========+=========+=========+
Ec-21_005630     PAVLVSASEVFEYLLDFRSAEIILLGCLLHDPDYKPALSSYGRLAARKGELSVAERYLTR
MSTRG.41586.1    ------------------------------------------------------------
                                                                             


                       1330      1340      1350      1360      1370      1380
                 =========+=========+=========+=========+=========+=========+
Ec-21_005630     ATSLSGGRDHAAKGGERGGKTAGVGAAEDWLTLMKFYAERTPSSARATDTLTCFRKVLSC
MSTRG.41586.1    ------------------------------------------------------------
                                                                             


                       1390      1400      1410      1420      1430      1440
                 =========+=========+=========+=========+=========+=========+
Ec-21_005630     LDWTNLEQQRVAQSLPRKATATAAPQGGVDSTTSPQSSSETFDKSWQPRGEILLELGRFE
MSTRG.41586.1    ------------------------------------------------------------
                                                                             


                       1450      1460      1470      1480      1490      1500
                 =========+=========+=========+=========+=========+=========+
Ec-21_005630     LVHNRSIKRASAFFRACLRESPWHASAKVHLAVCMLEQLGPFKDGRPPSRATMKIDSLLR
MSTRG.41586.1    ------------------------------------------------------------
                                                                             


                       1510      1520      1530      1540      1550      1560
                 =========+=========+=========+=========+=========+=========+
Ec-21_005630     SVLNFNGYGHVTRSRTPTLNTVGGNVRPKASAAPPSSPTLTPTRIRGKGASASVGATATT
MSTRG.41586.1    ------------------------------------------------------------
                                                                             


                       1570      1580      1590      1600      1610      1620
                 =========+=========+=========+=========+=========+=========+
Ec-21_005630     NHAGHNTPIKYDATPRSSSVGAARHGGGGGGGRGIWLLWLFYAKFAERCLKDPQLALFAL
MSTRG.41586.1    ------------------------------------------------------------
                                                                             


                       1630      1640      1650      1660      1670      1680
                 =========+=========+=========+=========+=========+=========+
Ec-21_005630     NRAAAAAASLPGRLGQYRSRRGLNPNGGTIGDGVKLDFRGGAVPFTLVARAQFCQRWPKL
MSTRG.41586.1    ------------------------------------------------------------
                                                                             


                       1690      1700      1710
                 =========+=========+=========+=======
Ec-21_005630     SECIAGSSGGGGDVDSSPEESLMDAAMRQAPLSEHYA
MSTRG.41586.1    ------------------------------------V
```

```
Parameters used
Minimum Number Of Sequences For A Conserved Position: 2
Minimum Number Of Sequences For A Flanking Position: 2
Maximum Number Of Contiguous Nonconserved Positions: 8
Minimum Length Of A Block: 10
Allowed Gap Positions: None
Use Similarity Matrices: Yes
```

```
Flank positions of the 4 selected block(s)
Flanks: [638  685]  [699  713]  [719  767]  [791  963]  

New number of positions in Ec-21_005630.aa.fasta.trans.aa_ali.fasta-gb:  285  (16% of the original 1717 positions)
```
